# Impact of kindergarten structures on the dynamics of hand, foot, and mouth disease and the effects of intervention strategies: an agent-based modeling study

**DOI:** 10.1186/s12916-025-04207-7

**Published:** 2025-07-01

**Authors:** Qing Zou, Xin-Fu Shi, Chang-Wei Liang, Meng-Meng Ma, Jing-Hua Li, Jing Gu, Ying-Si Lai

**Affiliations:** 1 Department of Medical Statistics, School of Public Health, Sun Yat-Sen University, Guangzhou, 510080 China; 2 Department of Infectious Diseases, Nanning Center for Disease Control and Prevention, Guangxi, 530023 China; 3 Department of Infectious Disease Prevention and Control, Guangzhou Center for Disease Control and Prevention, Guangzhou, 511430 China; 4 Sun Yat-sen Global Health Institute, Institute of State Governance, Sun Yat-Sen University, Guangzhou, 510080 China; 5 Faculty of Health Sciences, University of Macau, Macao SAR, 999078 China; 6 Institute of International and Regional Studies, Sun Yat-sen University, Guangzhou, 510080 China; 7 Health Information Research Center, Guangdong Key Laboratory of Medicine, School of Public Health, Sun Yat-Sen University, Guangzhou, 510080 China; 8 Guangzhou Joint Research Center for Disease Surveillance, Early Warning and Risk Assessment, Guangzhou, 510080 China

**Keywords:** Agent-based model, Hand, foot, and mouth disease, Kindergarten, Class size, Non-pharmaceutical interventions

## Abstract

**Background:**

Hand, foot, and mouth disease (HFMD) poses an unignorable threat to the health of kindergarten children. Kindergarten structures (i.e., class size and kindergarten size) may influence the transmission dynamics and the effectiveness of non-pharmaceutical interventions (NPIs), but few studies have explored these effects.

**Methods:**

We developed an agent-based network model to study the effects of kindergarten structures on dynamics of HFMD caused by three types of strains (i.e., EV-A71, CVA16, and other EVs). We pursued a systematic review to collect data on HFMD outbreaks to estimate key model parameters. We simulated a series of scenarios to study the effects of NPIs (i.e., isolation of symptomatic individuals, class and family quarantine, and kindergarten closure, organized stepwisely), under different kindergarten sizes (*n* = 180, 360, and 900) and class sizes (*m* = 10, 20, 30, 60, etc.). We further explored alternative interventions combined with vaccination to avoid kindergarten closure during an outbreak.

**Results:**

Overall, we found that the larger the class size, the more cumulative infections and the less effectiveness of NPIs in kindergartens. Stronger NPIs resulted in better effectiveness, and the variations in effectiveness among different class sizes gradually reduced with stronger interventions. Similar patterns were shown in kindergartens with small, medium, and large sizes. NPIs including kindergarten closure, which is implemented in many endemic countries, was a potent epidemic control strategy, capable of reducing cumulative incidence by over 80% for most class sizes in medium-size kindergartens. For EV-A71 infections, a vaccine coverage of 50% was alternative to kindergarten closure, when class size was 60 or less in medium-size kindergartens.

**Conclusions:**

Kindergarten structures, particularly class size, had an important impact on dynamics of HFMD and effectiveness of NPIs within kindergarten. Increasing vaccination coverage may be an alternative to kindergarten closure for control of the disease.

**Supplementary Information:**

The online version contains supplementary material available at 10.1186/s12916-025-04207-7.

## Background

Hand, foot, and mouth disease (HFMD) is a common acute viral infectious disease that poses a serious threat to the health of children under 5 years old [1]. Children are usually infected with HFMD through close contact with an infected person or their contaminated belongings. Around 90% of outbreaks occurred in kindergartens [2] making it the most common environment for the outbreaks of HFMD. Multiple studies have suggested that the structure of school population might influence the transmission dynamics of infectious diseases commonly found in children through close contact [3]. Children tended to make contacts with others of a similar age, a principle known as assortativity [4]. Contact frequencies with classmates tend to be higher than those with peers in different classes [5], which may elevate the risk of transmission within the class [6]. In addition, it has been found that the relative contribution of within-class and within-grade transmissions to the reproduction number varied with the number of classes per grade [7]. It is of profound and wide-reaching significance to understand how the school population structure influence the transmission dynamics.

There is no specific antiviral treatment for HFMD. Vaccination and non-pharmaceutical interventions (NPIs) are considered the commonly effective methods for prevention and control of HFMD [8]. NPIs mainly include isolation of symptomatic infections [9], suspending classes, and school closures [10], while in most endemic countries (e.g., China, Thailand, Singapore) the government-recommended control strategy is the combination of the above methods [11]. These NPIs aim to reduce the frequencies of contacts between individuals and disrupt potential chains of transmission. Studies have suggested that the effects of even the same interventions may vary across school population structures [12]. Yet, no study has quantified such differences under different population structures. Another concern is that school closure is low cost-effective and justifiable only in response to high-severity epidemics [13]. Minimizing unnecessary school closure to find a better balance between cost-effective, educational benefits, and outbreak control remains a major challenge for policy makers.

To fill the above knowledge gaps, in this paper we modeled HFMD transmission within kindergarten based on an individual-level contacting agent-based model, to answer the following two questions: (1) How do the kindergarten population structures (e.g., school size, class size) of kindergarten impact the HFMD transmission? (2) And how do they impact the effectiveness of NPIs? Furthermore, we explored the possible strategies to avoid kindergarten closure during a HFMD outbreak.

## Methods

### Method overview

Inspired by a SEIR model developed by Giardina and colleagues [14], we developed an age-specific, structurally explicit, agent-based network model to simulate HFMD epidemic within kindergarten. The overall transmission probability per contact per day was calibrated based on kindergarten outbreak data collected from literature review, using Bayesian methods. For individual contacts, the transmission risk was further weighted according to the different settings, such as contact place (e.g., classroom, home), sex, age, and whether or not they have symptoms. We simulated a series of scenarios to evaluate the outbreak sizes and the effects of control strategies under different kindergarten sizes and class sizes. In particular, we considered the following control strategies: (1) strategies consisted of the three common NPIs (i.e., isolation of symptomatic individuals, class/family quarantine, and kindergarten closure) with a “stepwise” inclusion process; (2) vaccination with different rates additional to the above strategies. Sensitivity analyses were undertaken afterwards.

We followed the Overview, Design concepts, and Details (the first section of Additional file 1) (ODD) protocol [15] to show the details of the agents and parameters.

### Procedures

#### Modeled population and connect network

**Fig. 1:**
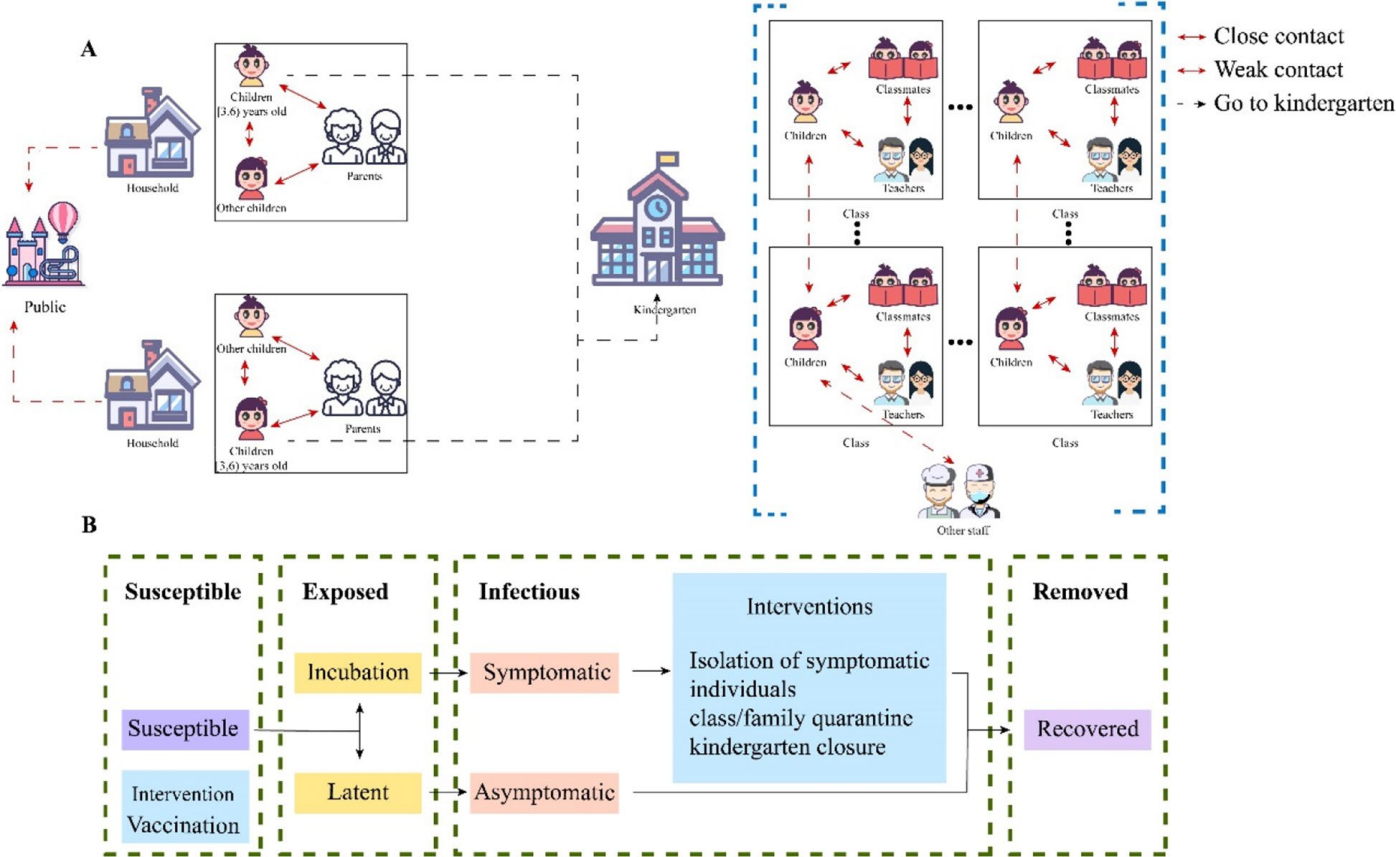
Model structure. **A** Contact diagram; **B** transmission model and interventions

We constructed a synthetic population with children, teachers, and other kindergarten staff in the kindergarten and other related people in households (e.g., children’s siblings and parents) (Fig. 1A). The synthetic population matches key demographic measures according to the China Statistical Yearbook 2016, including age structure, sex ratio, the distribution of household size, and family contact matrix. Transmission in the model occurs at three contact layers (kindergarten, household, and place for public activities) through connect network (Fig. 1A and connect network section, Additional file 1).

#### Transmission model

Briefly, within the transmission model, individuals, denoted as agents, are modeled as either susceptible to the virus, exposed to it, infected, or recovered. In addition, infected and infectious agents are categorized as either asymptomatic or symptomatic (Fig. 1B). We assumed that a HFMD-infected individual $i$ transmits to a susceptible one $j$ at time-step $t$ with a probability ${p}_{ijkt}={{\beta }_{0}c}_{ijkt}{q}_{k}{s}_{j}{r}_{i}{{g}_{i}{g}_{j}a}_{i}{w}_{T}$ (1). Here, ${\beta }_{0}$ indicates the baseline of transmission rate, the key estimand in the model, and other parameters (i.e., ${c}_{ijkt},{q}_{k},{s}_{j},{r}_{i},{{g}_{i},{g}_{j},a}_{i},{w}_{T}$) were used to adjust the influence of gender, age, layers of contact, seasonality, and other factors on the transmission rate (see “Transmission rate” section in Additional file 1).

#### Interventions

Four interventions, including three NPIs (i.e., isolation of symptomatic individuals, class and family quarantine, and kindergarten closures) and vaccination, were considered in the model (see “Intervention” section in Additional file 1).

#### Parameter estimation

The model comprised parameters in two aspects: disease transmission parameters (including relative probabilities of state transitions and time to disease progression) and intervention-related parameters. Most of the parameters (e.g., age-specific susceptibility, gender-related multiplier, and incubation period) were derived from published studies (Additional file 1: Table S1).

To estimate the key unknown parameter—the baseline transmission rate (i.e., ${\beta }_{0}$ in formula (1)), we searched for relevant publications pertaining to outbreaks of HFMD in a kindergarten-based or a school-based setting through a systematic review (registered in the International Prospective Register of Systematic Reviews, PROSPERO, No. CRD42022377235 [16], and see Supplementary information). We employed the Sampling Importance Resampling algorithm to infer the posterior distribution of ${\beta }_{0}$, as it efficiently handles complex posterior distributions [17, 18]. To assess model performance, we compared whether the actual attack rate was within the 95% BCI of the predicted value as well as calculated the mean absolute percentage error (MAPE) [19] and correlation coefficient ($r$) (see “Model validation” section in Additional file 1).

#### Simulation scenarios

We simulated the occurrence of HFMD caused by three types of strain (i.e., EV-A71, CVA16, and other EVs) in three sizes of kindergartens (small-size: *n* = 180, medium-size: *n* = 360, and large-size: *n* = 900) with different class sizes (small-size: *m* = 10, 20, or 30; medium-size: *m* = 40, 50, or 60; large-size: *m* = 100 or 120). The population structure of kindergartens of different sizes is shown in Table 1. To begin a simulation, we randomly designated one of the kids in kindergarten as infected person, i.e., an indicator case, starting on a random day of a week. The model was then run for 30 days to allow sufficient time for the duration of an outbreak taking place within the kindergarten. Simulation scenarios are as follows:

1. No interventions (baseline scenario). We set no interventions (L0) in the baseline scenario, to explore the net effect of kindergarten structures on the transmission of HFMD.
2. Stepwise NPIs. We combined the three single NPI considered in the model (see “Intervention” section in Additional file 1) using a “stepwise” approach to form intervention strategies with different intensities. L1: only isolation of symptomatic individuals; L2: combination of isolation of symptomatic individuals, class quarantine, and family quarantine (i.e., when there are more than two symptomatic individuals in the same family or class, the family or class will be isolated); L3: combination of the three NPIs, including isolation of symptomatic individuals, class/family quarantine, and kindergarten closure (i.e., when there are more 10 cases in the whole kindergarten within a week, or there are more than 2 cases in three classes, the whole kindergarten will be suspended for 10 days), which is the common governmental recommended NPIs strategies in childcare setting.
3. Explore alternative strategies for kindergarten closure. To explore alternative strategies instead of kindergarten closure, we further combined vaccination with different levels of coverage (V0: none, 0%; V1: low-level, 20%; V2: medium-level: 50%; V3: high-level, 80%) into the above strategies, resulting in a total of 16 scenarios (Table 2). As only the inactivated EV-A71 vaccine has been successfully approved for marketing as an HFMD-associated enterovirus vaccine, we only consider EV-71 in this case. To simplify the process, we only simulated the scenarios in medium-sized kindergarten.

**Table 1:** Different population structures of kindergarten

Kindergarten sizes	No. grades	Class sizes (*m*)	No. classes per grade
Small (*n* = 180)	3	10	6
		20	3
		30	2
		60	1
Medium (*n* = 360)	3	10	12
		20	6
		30	4
		40	3
		60	2
		120	1
Large (*n* = 900)	3	10	30
		20	15
		30	10
		50	6
		60	5
		100	3

Data are *n*. Small-size (*m* = 10 or 20), medium-size (*m* = 30, 40, or 50), large-size (*m* = 60, 100, or 120)

**Table 2:** Combination of stepwise NPIs and vaccination

NPIs strategies	Vaccine coverage rate			
	V0	V1	V2	V3
L0	L0 + V1	L0 + V1	L0 + V2	L0 + V3
L1	L1 + V1	L1 + V1	L1 + V2	L1 + V3
L2	L2 + V1	L2 + V1	L2 + V2	L2 + V3
L3	L3 + V1	L3 + V1	L3 + V2	L3 + V3

*Abbreviations*: *NPIs* non-pharmaceutical interventions, *L0* baseline with no intervention, *L1* only isolation of symptomatic individuals, *L2* combination of isolation of symptomatic individuals and class and family quarantine, *L3* combination of three NPIs, including isolation of symptomatic individuals, class and family quarantine, and kindergarten closure (governmental recommended NPIs strategies in child care setting), *V0* vaccine coverage rate equals 0%, *V1* vaccine coverage rate equals 20%, *V2* vaccine coverage rate equals 50%, *V3* vaccine coverage rate equals 80%

### Statistical analysis

To balance computational efficiency with robust statistical analysis, we simulated each scenario under ten random number seeds [20, 21] and summarized the results. We calculated the cumulative number of infections (including asymptomatic infections) and the effective reproduction number (${R}_{t}$) per day to measure the outbreak and the transmission dynamics, respectively. The effects of NPIs strategies were quantified by calculating the proportion of reduced cumulative incidence, using the following formula: attributable percentage (${AP}_{i}$) = $\frac{{\rho }_{0}-{\rho }_{i}}{{\rho }_{0}}\times 100\%$, where ${\rho }_{0}$ and ${\rho }_{i}$ denote the cumulative infection rate of baseline scenario and $i$ scenario, respectively. We used the government-recommended NPIs (L3) [11] as the reference scenario and calculated the infection rate ratio (IRR) to evaluate the relative effect of each intervention strategy compared to L3. IRR with 95% Bayesian credible interval (BCI) less than 1 is considered to indicate an intervention effect that is superior to the effectiveness of the government-recommended intervention strategy, while 95% BCI of IRR including 1 is considered to be equivalent.

In the above scenarios, the duration of school closure ($t=10\;\text{days}$) and the application prerequisites of school closure (i.e., the number of symptomatic individuals, *n* = 10) are set based on the government-recommended strategy in China. However, the details of the implementation of interventions slightly vary cross different countries. Thus, we simulated scenarios with different duration of school closure ($t=5 \text{days}, t=7 \text{days}, t=10 \text{days}$) and application prerequisites of school closure (i.e., the number of symptomatic individuals *n* = 5, *n* = 7, *n* = 10) to explore whether these factors would have an impact on the results. What is more, we varied the seasons (i.e., high incidence risk period from May to July and low incidence period from January to February) to assess the reliability of the outcomes.

All analyses were implemented in R version 3.6.3.

## Results

### Parameter estimation and model validation

Through systematically reviewing peer-reviewed literature and subsequent literature screening, 23 records were included. These included 12 articles related to EV-A71, 9 articles related to CVA16, and 2 articles related to other EVs (Additional file 1: Fig. S2). The basic transmission rates of EV-A71, CVA16, and other EVs were estimated 0.0087 (95% BCI: 0.0061, 0.0107), 0.0115 (95% BCI: 0.0095, 0.0143), and 0.0190 (95% BCI: 0.0146, 0.0219), respectively (Additional file 1: Table S2). Model validation suggested the model with good performance, as the actual values of both the EV-A71-related and CVA16-related training sets were within 95% BCI of the predicted values, as well as the $\text{MAPE}$ < 20% and $r$ > 0.9 (Additional file 1: Table S3).

### Evaluating the impact of class size on disease transmission of HFMD

**Fig. 2:**
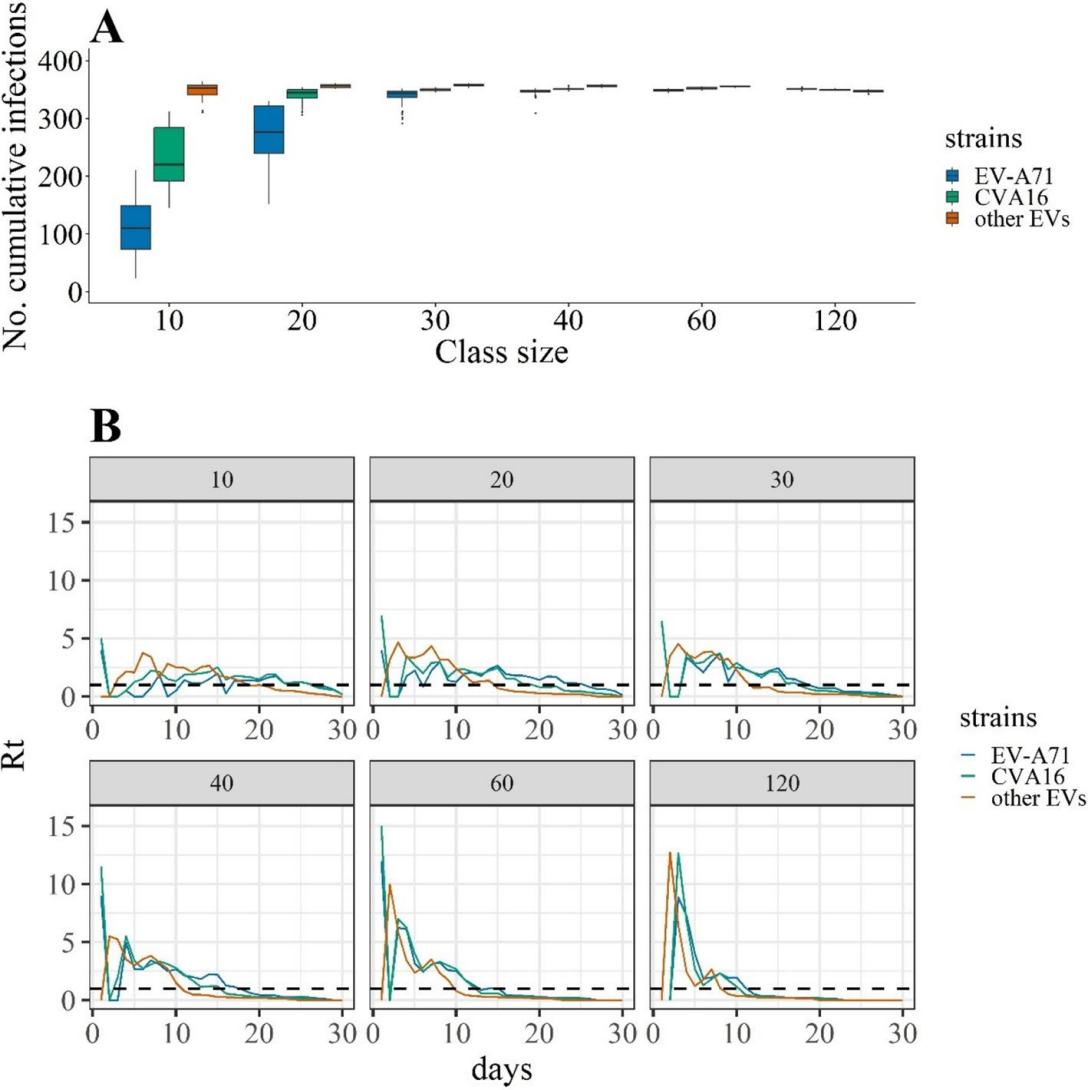
The transmission dynamics of hand, foot, and mouth disease in the baseline scenario in a medium-size kindergarten. **A** The cumulative number of infections caused by EV-A71, CVA16, and other EVs. **B** The time series plot of the effective reproductions (*R*_*t*_) per day caused by EV-A71, CVA16, and other EVs. Dashed line indicates *R*_*t*_ = 1

In the baseline scenario, for the three types of strains (EV-A71, CVA16, and other EVs) and in a medium-sized kindergarten, larger the class sizes caused more cumulative infections, when the class sizes were less than 30; when class sizes were greater than 30, the cumulative infections increased less, as most susceptible individuals got infection, without any intervention (Fig. 2A). The time series plots for ${R}_{t}$ of the three types of strains showed that as the class size increased, ${R}_{t}$ showed higher in the early phase. However, the decline speed of ${R}_{t}$ seems inversely to class size: when the class size was large, ${R}_{t}$ decreased more rapidly, suggesting more individuals got infection in early phase; when the class size was small, ${R}_{t}$ decreased slowly, with ${R}_{t}$ below 1 slower (Fig. 2B).

Kindergartens with small or large sizes showed similar patterns that larger the class sizes caused more cumulative infections when the class sizes were less than 30, suggesting that the kindergarten size seemed affect little on the effect of class size pattern on transmission. However, with the increase of kindergarten size, the total cumulative number of infected individuals increased (Fig. 2 and Additional file 1: Figs. S3 and S4).

### Evaluation of the impact of class size on the effectiveness of NPIs strategies

**Fig. 3:**
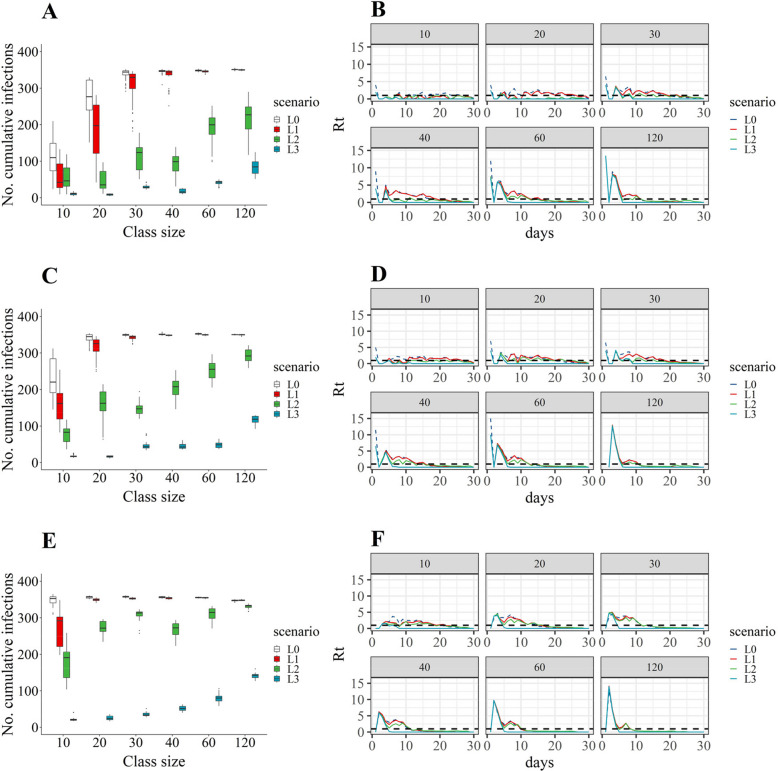
The transmission dynamics of hand, foot, and mouth disease in medium-size kindergarten under three intervention strategies. **A**, **C**, and **E** The cumulative number of infections caused by EV-A71, CVA16, and other EVs, respectively. **B**, **D**, and **F** The time series plot of the effective reproductions (*R*_*t*_) per day caused by EV-A71, CVA16, and other EVs, respectively. L0: baseline with no intervention; L1: only isolation of symptomatic individuals; L2: combination of isolation of symptomatic individuals and class and family quarantine; L3: combination of three NPIs, including isolation of symptomatic individuals, class and family quarantine, and kindergarten closure (governmental recommended NPIs strategies in child care setting). Dashed line indicates *R*_*t*_ = 1

The intensity of the NPIs strategies was progressively stronger from L1 to L3. Larger class sizes resulted in higher number of cumulative infections. Particularly, L1 strategy, isolation of symptomatic individuals alone, only shown moderate or minor effects with small class sizes (*m* < 30) in medium-size kindergartens (Fig. 3). L2, adding the measure of class and family quarantine, had better effects. For instance, in a class of 30 students, they could reduce the number of infections caused by EV-A71, CVA16, and other EVs by 63.81% (95% BCI: 49.63%, 83.48%), 57.89% (95% BCI: 47.90%, 64.96%), and 12.13% (95% BCI: 10.91%, 13.62%), respectively, in medium-size kindergartens (Table 3). Still, large number of cumulative infections was resulted when class sizes were large (*m* ≥ 60).

**Table 3:** Reduced cumulative incidence by NPIs strategies in medium-size kindergartens

Class sizes	Scenarios	EV-A71	CVA16	Other EVs
		**Attributable percentages (%)**	**Attributable percentages (%)**	**Attributable percentages (%)**
		**Median (95% BCI)**	**Median (95% BCI)**	**Median (95% BCI)**
**10**	**L0**	[Reference]	[Reference]	[Reference]
	**L1**	53.28 (32.53–69.37)	31.47 (22.46–42.58)	19.56 (16.15–35.76)
	**L2**	51.84 (32.63–66.09)	64.73 (56.92–73.55)	45.99 (41.28–59.33)
	**L3**	89.30 (73.40–94.33)	91.63 (88.59–95.00)	94.85 (94.60–95.06)
**20**	**L0**	[Reference]	[Reference]	[Reference]
	**L1**	33.48 (16.57–64.24)	8.28 (1.72–22.87)	1.99 (1.53–2.87)
	**L2**	85.88 (71.09–91.19)	53.50 (41.42–78.90)	22.43 (18.85–25.65)
	**L3**	96.87 (96.56–97.21)	95.79 (94.87–97.01)	94.67 (93.02–95.06)
**30**	**L0**	[Reference]	[Reference]	[Reference]
	**L1**	5.79 (2.02–36.54)	3.84 (1.83–7.78)	1.07 (0.46–2.00)
	**L2**	63.81 (49.63–83.48)	57.89 (47.90–64.96)	12.13 (10.91–13.62)
	**L3**	93.39 (87.87–94.19)	90.63 (83.42–92.29)	92.73 (91.82–93.06)
**40**	**L0**	[Reference]	[Reference]	[Reference]
	**L1**	1.74 (− 2.04–19.02)^a^	0.53 (− 1.31–2.51)^a^	0.69 (0.02–1.33)
	**L2**	71.42 (60.32–88.85)	41.09 (28.30–54.48)	23.34 (20.87–27.32)
	**L3**	96.18 (93.81–97.10)	90.52 (86.96–91.86)	89.21 (88.07–90.22)
**60**	**L0**	[Reference]	[Reference]	[Reference]
	**L1**	1.36 (− 0.57–4.28)^a^	0.75 (− 1.70–2.90)^a^	1.27 (0.28–1.66)
	**L2**	41.30 (29.89–67.62)	26.64 (17.61–40.85)	12.20 (10.55–16.17)
	**L3**	90.24 (89.17–93.46)	89.50 (86.17–91.96)	83.21 (81.64–84.18)
**120**	**L0**	[Reference]	[Reference]	[Reference]
	**L1**	0.29 (− 1.76–1.66)^a^	0.25 (− 2.21–2.29)^a^	− 0.77 (− 1.85 to − 0.16)
	**L2**	34.76 (20.85–63.16)	16.38 (7.78–24.33)	6.56 (6.16–7.62)
	**L3**	82.61 (73.57–88.44)	75.81 (72.93–80.51)	71.24 (70.59–72.95)

*Abbreviations*: *EV-A71* enterovirus A71, *CVA16* coxsackievirus A16, *Other EVs* other enterovirus, *L0* baseline with no intervention, *L1* only isolation of symptomatic individuals, *L2* combination of isolation of symptomatic individuals and class and family quarantine, *L3* combination of three NPIs, including isolation of symptomatic individuals, class and family quarantine, and kindergarten closure (governmental recommended NPIs strategies in child care setting)

^a^Difference not significant, with 95% BCI including zero

L3, combination of the three NPIs, was a potent epidemic control strategy, capable of reducing cumulative incidence by over 80% for most class sizes (Table 3). Besides, L3 strategy seemed less affected by class size. Even with larger class sizes (*m* ≥ 60), obvious reduction (> 70%) of cumulative incidence was shown (Fig. 3 and Table 3).

Overall, the outcomes across kindergartens of different sizes (i.e., small, medium, and large) showed a similar pattern: the number of cumulative infections increased as the class size increased (Fig. 3 and Additional file 1: Figs. S5 and S6), and with escalated intervention intensities (from L1 to L3), the variations in effectiveness among different class sizes gradually reduced (Table 3 and Additional file 1: Tables S3 and S5).

### Exploring alternative strategies for kindergarten closure

Specific to EV-A71, there were certain intervention strategies combined vaccination, with effects superior or equivalent to the government-recommended NPIs strategies. Results showed the trend that the smaller the class sizes, the lower vaccine coverages were required. For class size equaling to 10, strategies with vaccination rates of 20% or above, combined with either L1 or L2 without kindergarten closure, can be as effective as the NPIs recommended by the government with kindergarten closure. When class sizes ranged from 20 to 60, comparable results can be achieved with the vaccination rate of 50%. When class sizes exceeded 60, higher vaccination rates, e.g., 80%, were required (Table 4).

**Table 4:** Relative effects of intervention strategies in medium-size kindergarten against EVA-71^a^

Class sizes	NPIs strategies	Vaccine coverage rate			
		**V0**	**V1**	**V2**	**V3**
		**IRRs**	**IRRs**	**IRRs**	**IRRs**
		**Median (95% BCI)**	**Median (95% BCI)**	**Median (95% BCI)**	**Median (95% BCI)**
**10**	**L0**	9.34 (3.76–17.65)	4.98 (1.84–7.68)	0.98 (0.47–2.20)^b^	0.33 (0.19–0.39)^c^
	**L1**	4.07 (1.67–11.21)	1.87 (0.66–4.18)^b^	0.33 (0.23–1.01)^b^	0.23 (0.17–0.34)^c^
	**L2**	4.25 (1.71–10.29)	0.61 (0.26–1.72)^b^	0.48 (0.23–1.20)^b^	0.22 (0.17–0.34)^c^
	**L3**	[Reference]	0.43 (0.24–0.83)^c^	0.27 (0.19–0.51)^c^	0.21 (0.16–0.34)^c^
**20**	**L0**	31.96 (29.03–35.84)	18.37 (11.36–27.43)	6.89 (2.95–11.90)	0.70 (0.57–0.94)^c^
	**L1**	21.16 (12.26–29.44)	9.61 (4.43–13.10)	2.21 (1.24–6.44)	0.64 (0.41–1.10)^b^
	**L2**	4.48 (3.05–10.20)	4.80 (2.67–6.84)	1.43 (0.89–3.93)^b^	0.51 (0.41–0.64)^c^
	**L3**	[Reference]	2.22 (1.63–3.03)	1.10 (0.76–1.88)^b^	0.49 (0.38–0.57)^c^
**30**	**L0**	15.13 (8.24–17.20)	11.21 (5.33–14.59)	4.84 (1.44–7.88)	0.68 (0.25–1.22)^b^
	**L1**	13.52 (5.16–16.23)	8.70 (3.36–12.91)	2.66 (0.64–6.82)^b^	0.34 (0.10–0.64)^c^
	**L2**	4.77 (1.61–7.83)	3.10 (1.17–5.50)	0.60 (0.13–1.87)^b^	0.24 (0.10–0.38)^c^
	**L3**	[Reference]	0.40 (0.19–0.53)^c^	0.47 (0.18–0.85)^c^	0.14 (0.09–0.19)^c^
**40**	**L0**	26.18 (16.17–34.49)	20.77 (14.31–28.29)	10.36 (8.49–14.11)	1.34 (0.77–1.78)^b^
	**L1**	24.01 (16.29–33.47)	16.06 (12.83–23.25)	6.83 (2.67–9.14)	0.48 (0.29–0.77)^c^
	**L2**	6.34 (4.52–9.71)	4.87 (3.63–7.00)	2.04 (0.70–2.81)^b^	0.32 (0.19–0.48)^c^
	**L3**	[Reference]	0.77 (0.56–1.33)	1.11 (0.61–1.73)^b^	0.21 (0.14–0.27)^c^
**60**	**L0**	10.25 (9.23–15.29)	9.15 (8.37–13.23)	6.95 (5.90–7.95)	1.92 (1.03–3.22)
	**L1**	10.17 (9.04–15.06)	8.87 (7.97–12.45)	5.98 (4.80–6.68)	0.83 (0.35–1.61)^b^
	**L2**	6.16 (3.39–6.95)	3.73 (2.72–4.47)	1.40 (0.68–1.80)^b^	0.28 (0.10–0.57)^c^
	**L3**	[Reference]	0.59 (0.47–0.67)^c^	0.37 (0.32–0.49)^c^	0.15 (0.09–0.18)^c^
**120**	**L0**	5.75 (3.79–8.65)	5.31 (3.59–7.92)	4.87 (3.34–6.73)	3.68 (2.78–4.18)
	**L1**	5.66 (3.79–8.58)	5.15 (3.55–7.71)	4.66 (3.12–6.16)	2.29 (1.81–2.77)
	**L2**	3.66 (2.78–4.24)	2.86 (2.04–3.39)	1.31 (1.01–1.73)	0.33 (0.25–0.51)^c^
	**L3**	[Reference]	0.76 (0.56–1.00)^c^	0.50 (0.34–0.59)^c^	0.19 (0.15–0.26)^c^

*Abbreviations*: *NPIs* non-pharmaceutical interventions, *L0* baseline with no intervention, *L1* only isolation of symptomatic individuals, *L2* combination of isolation of symptomatic individuals and class and family quarantine, *L3* combination of three NPIs, including isolation of symptomatic individuals, class and family quarantine, and kindergarten closure (governmental recommended NPIs strategies in child care setting), *V0* vaccine coverage rate equals 0%, *V1* vaccine coverage rate equals 20%, *V2* vaccine coverage rate equals 50%, *V3* vaccine coverage rate equals 80%

^a^Relative effects of intervention strategies were measured by infection rate ratios (IRR), which were calculated by dividing the incidence rate of each scenario by that of the reference scenario for the same class size

^b^Equivalent effect with 95% BCI including one

^c^Superior effect with 95% BCI less than one

### Sensitivity analyses

Sensitivity analyses showed that under equivalent class size, the cumulative number of infections was relatively higher with shorter durations of school closure or more lenient application prerequisites (i.e., school closures are implemented as more symptomatic people are found). Nevertheless, these two factors affected less the trend of the effects of class sizes on the transmission (Additional file 1: Fig. S7A, B). Likewise, adjustments in seasonality did not wield any substantial influence on the overall trend (Additional file 1: Fig. S7C).

## Discussion

This study contributed to the prevention and control of HFMD in kindergarten in two aspects. First, to our knowledge, this is the first study to explore the potential impact of class size on the dynamics of HFMD and the effectiveness of NPIs in kindergarten, by developing an agent-based model of HFMD. Second, we explored potential alternative strategies to control the spread of HFMD without kindergarten closure, which provide insights for future epidemic prevention and control. The adopted approach can be applied in other kindergarten settings or under other similar contact-transmitted diseases to understand the interaction between the population structures and dynamics of disease spread. Our study framework also provides the flexibility to study diverse scenarios, allowing researchers to adjust the parameters specific to other transmission settings for potential future pandemics.

Our study found that the smaller the class size, the lesser the severity of the epidemic of HFMD in a kindergarten, which is compatible with previous studies on other infectious disease, such as dengue fever [22], COVID-19 [23], and influenza. A more nuanced result of our analysis indicated that control strategies targeting smaller class size rather than the large class size are more effective, highlighting the importance of small class size for epidemic prevention and control. Thus, class size for epidemic prevention and control should be considered when planning future kindergarten class structures. We found that by implementing only isolation of symptomatic individuals and class/family quarantine, once the class size reaches 30 (which is typical in Chinese kindergartens), most children may become infected and the intervention had minimal effect. Even with smaller class sizes, a higher percentage of children could still be infected. Therefore, it is necessary to implement stronger HFMD interventions in kindergartens. Here, our results suggested that the combination of NPIs (i.e., isolation of symptomatic infections, suspending classes, and school closures), implemented in China and many other endemic countries, was clearly successful in mitigating spread and reducing local transmission of HFMD. This finding aligns with a significant amount of literature that acknowledges school closures as an effective NPI [24]. Besides, the above findings are consistent across the three analyzed strains: EV-A71, CVA16, and others EVs. The estimated parameter showed that the infectivity of EV-A71 and CVA16 was no significant difference, while that of other EVs was slightly higher, which was consistent with other studies’ results [25]. The difference in infectivity also resulted in prevention and control measures being less effective against other EVs than EV-A71 and CVA16.

Despite the efficacy of school closure in controlling the spread of HFMD, potential economic and educational losses are highlighted [26]. After considering the balance between socioeconomic benefits and health impacts, our results suggested that the deployment of vaccine could be a viable alternative to school closure [27, 28]. The current National Immunization Program vaccine in China does not include the monovalent EV-A71 vaccine, and as of the end of 2021, the national average cumulative vaccination rate stands at only 24.96% [29], which is far below the requirement of herd immunity [30]. Therefore, it is recommended to increase the vaccination coverage of the key population to reduce their risk of infection. On the other hand, only EV-A71 vaccination was approved by National Medical Products Administration for preventing HFMD [31]. Therefore, we only modeled the scenario of vaccination against EV-A71, and our results can only suggest that increasing vaccination rates could be an alternative to school closure in controlling HFMD caused by EV-A71. However, several researchers have found that the proportion of EV-A71-caused HFMD decreased with the popularization of EV-A71 vaccination, while the proportion of HFMD caused by other serotypes such as CVA16 and CVA10 increased [32]. This suggests multivalent vaccines and more effective vaccination strategies different from routine inoculation, such as pulse vaccination, would play an important role in preventing the transmission of all-type HFMD in the future. Currently, the inactivated polyvalent vaccines, including bivalent, trivalent, and quadrivalent vaccines, have primarily been tested for their protective effects in animal studies, lacking clinical evidence of protection [31]. Further efforts are needed. Although such analyses were beyond the scope of this paper, we are planning to explore this in future work.

### Limitations

Frankly, there are several limitations in our study. Firstly, to simplify the model, we only considered transmission within the kindergarten and excluded the influence of introduced infections from outside, which is in line with common real situation. This might partially affect the overall number of infections, but seemed had little impact on the main findings. Secondly, we estimated the basic transmission parameters separately for only EV-A71 and CAV16, the two most common causative strains of HFMD. For other causative strains, including those with increasing outbreaks such as coxsackievirus A6 (CVA6) and coxsackievirus A10 (CVA10) [32], due to the unavailability of sufficient data, we grouped them under the “other EVs” category to estimate an overall basic transmission parameter. This underscores the urgent need for more detailed data. Incorporating separate estimates of CVA6 and CVA10 will be a direction for our future research to better guide the development of current prevention and control measures. Finally, it should be noted that simulation results from agent-based models are challenging to validate, despite the parameters having been calibrated and estimated based on a comprehensive review of the relevant literature. Real-life experimentations for validation are important, which could be a key focus of future research.

## Conclusions

Kindergarten structures, particularly class size, had an important impact on dynamics of HFMD and effectiveness of NPIs within kindergarten, which should be emphasized by policy makers to develop more flexible and targeted interventions. Increasing vaccination coverage provides large beneficial, and the development of multivalent vaccines is important for control and prevention of multiple types of HFMD in the future.

## Supplementary Information

Additional file 1. Composed of three sections. The first section provides a detailed description of the model according to the ODD protocol. The second section serves as a supplement to the methodology, while the third section presents additional results [33–58].

## Data Availability

No datasets were generated or analysed during the current study.

## Abbreviations

BCI: Bayesian credible interval
HFMD: Hand, foot, and mouth disease
IRR: Infection rate ratio
MAPE: Mean absolute percentage error
NPIs: Non-pharmaceutical interventions
