# Supplementary material for: Impact of kindergarten structures on the dynamics of hand, foot, and mouth disease and the effects of intervention strategies: an agent-based modeling study
Source: BMC Med. 2025 Jul 1;23:357. doi: 10.1186/s12916-025-04207-7 (PMC12211278; doi:10.1186/s12916-025-04207-7)
Supplement: Supplementary file 1 — Additional file 1. Composed of three sections. The first section provides a detailed description of the model according to the ODD protocol. The second section serves as a supplement to the methodology, while the third section presents additional results [33–58]. [file 12916_2025_4207_MOESM1_ESM.docx]

**Supplemental Materials**

**Impact of kindergarten structures on the dynamics of hand foot mouth disease and the effects of intervention strategies: an agent-based modelling study**

**Supplementary information**

**Fig. S1.** The fitting results of relative risk of transmission

**Fig. S2.** Data search and selection flow chart

**Fig. S3.** The transmission dynamics of hand, foot and mouth disease in small size kindergarten

**Fig. S4.** The transmission dynamics of hand, foot and mouth disease in large size kindergarten

**Fig. S5.** The transmission dynamics of hand, foot and mouth disease in small size kindergarten under three intervention strategies

**Fig. S6.** The transmission dynamics of hand, foot and mouth disease in large size kindergarten under three intervention strategies

**Fig. S7.** Cumulative number of infections under different parameter Settings

**Table S1** List of model parameters

**Table S2** Results of parameter estimation

**Table S3** Results of model validation

**Table S4** Reduced cumulative infections by NPIs strategies in small kindergartens

**Table S5** Reduced cumulative infections by NPIs strategies in large kindergartens

**Table S6** Attributes of agents

**Table S7** Information about the three contact layers

**Table S8** Summary of EV-A71-related and CVA16-related survey data of HFMD outbreaks

**Table S9** The result of quasi-Poisson regression

**Table S10** The triangular distribution parameter of relative risk of transmission

**Supplementary information**

**1. ODD protol description**

**Overview**

**Purpose**

We developed an age-specific, structurally explicit, agent-based network model to simulate HFMD epidemic in kindergarten. The purpose of the model was to understand how kindergarten population structures (e.g., class and school sizes) shape transmission dynamics of HFMD and how these affect the effectiveness of interventions.

**Entities,** **state variables, and scales**

We constructed a HFMD transmission scenario with kindergarten students as the core population, and incorporated interactions within schools and homes, as well as those between households outside school.

Entities: The number of agents in the simulation is mainly determined by the structure of the kindergarten population, and there are five types of agents: students, siblings, parents, teacher, staff. Each agent is described by some attributes (Table S6) and current infection state (Fig. S1).

State variables: Our disease model follows the SEIR framework, and the health states of each agent are characterized as follows:

- Susceptible: The agent is able to catch the virus.
- Exposed: The agent has caught the virus but is not yet infectious until late incubation period.
- Asymptomatic: The agent is infectious but not symptomatic.
- Symptomatic: The agent is infectious and symptomatic
- Recovered: The agent has survived the disease and is no longer infectious.

The model is visualized in Fig. 1B, where arrows illustrate possible state transitions. The different states of an infectious disease and their duration are effective parameters for simulating its spread.

Scales: The model runs at a 1-day time step, and was set for each person to have only one contact with others per day per setting. The model assumes all events occur during the day; it was run for a total of 30 days because HFMD is an acute infectious disease, most outbreaks end within a month [33].

**Process overview and scheduling**

The model proceeds in day steps, where agent infection status is updated. We assume that all individuals are initially susceptible, and that individual susceptibility to exposure is determined by individual-to-individual contact. The transition of the remaining states is determined by the probability and time parameters, as shown in Table S1. We do not consider the mortality state, since in real life the mortality rate of HFMD is close to 0 [34].

**Design concepts**

Emergence

Population dynamics emerge from the behaviour of the individuals, but the individual’s disease status and behaviour are entirely represented by empirical rules describing. For example, transmission rate and time parameters are represented as probabilities or probability distributions. Adaptation and fitness-seeking are thus not modelled explicitly, but are included in the empirical rules.

Sensing

Individuals do not have any knowledge of others’ infection status.

Interaction

Interactions of four contact layers are modelled:

- In the classroom, student-to-student, student-to-teacher, and teacher-to-teacher contact, where each person in the class is a contact for the other;
- In the school, student, teacher, and staff contact with each other, and each randomly contacting 20 individuals;
- At household, mutual contact among all family members;
- And outside the household, random family-to-family contact, with each family randomly contacts two families at a time.

Stochasticity

The model includes several stochastic processes related to the establishment of synthetic population, initialization of infection, inter-individual contact, and the change of health status. All behavioural parameters and time parameters are interpreted as probabilities, or are drawn from empirical probability distributions.

Observation

For each parameter set, the model was run 10 times. For model analysis, all state variables can be displayed and saved during each time step. We mainly collected the number of daily incidences, the number of daily symptomatic, and the number of infections at each contact level. And based on the results, we calculate disease indicators such as attack rate, cumulative number of cases, and effective reproduction number.

**Details**

Initialization

We assume that all people are initially susceptible, but susceptibility is age-specific, with children younger than 0-6 years being more susceptible. To begin a simulation, we randomly designated 1 member of the student population as infected, i.e., an indicator case, starting on a random day of the week. We also set the first day of the model start as a working day.

Input

1. The basic transmission rates are used as model input parameters and their values are obtained by parameter estimation.
2. The integrated population of participants in the simulation is generated in advance before the model is run, see below for details.
3. Since the duration of the health state obeys a probability distribution, the time parameter of each individual in the model is randomly sampled from the corresponding probability distribution.

**Synthetic population**

We generated a set of synthetic population through process steps which were as follows:

1. Synthetic kindergarten populations. The total number of kindergarten children aged [3-6) years was first determined as $w_{s}$, and we set there were 3 grades, $n$ classes per grade, $m$ children and 2 teachers per class in the kindergarten. We also incorporated $l$ additional staff to reflect such roles as administrators, counselors, cafeteria staff, custodians, and so on.
2. Synthetic family – assigns families and family members. First, according to the age structure of the Chinese population [35], we estimate the total number of children aged [0, 17] years based on the number of children aged [3-6) years, and assigned an age to each individual. Secondly, the number of different types of households were determined by the data on the distribution of families with children [36]. And then we randomly assigned a reference child and parents to every household. Finally, to construct the other child for households with more than one child, we assign siblings to the reference child from the remaining children with contact matrix as the sampling probability [37].

Excluded the households without kindergarten children (this part of the family is not associated with kindergarten and is not considered in the model).

**Connect network**

Transmission in the model occurs at three contact layers (Table S7). In kindergarten, students, teachers, and other staff interacted with each other; at home, children had randomized contact with siblings and parents; outside of household, students interacted with family members and other households (simulated public playgrounds or shared child care) [38]. And at the end of each time step, the data used for analysis are recorded.

**Transmission rate**

At each daily time-step, we modeled dyadic interactions between individuals according to household, classroom, school, and childcare relationships, drawing parameter values from the distributions or fixed value specified in Table S1. A HFMD-infected individual $i$ transmitted to susceptible individual $j$ at time-step $t$ with Bernoulli probability equal to $p_{ijkt}={\beta_{0}c}_{ijkt}q_{k}s_{j}r_{i}{g_{i}g_{j}a}_{i}w_{T}$ (1), where $\beta_{0}$was the baseline of transmission; $c_{ijkt}$ was an indicator variable equal to 1 if individuals $i$ and $j$ had contact type $k$ at time $t$, otherwise equal to 0; $q_{k}$ was the probability of transmission given one day of contact type $k$($k=$ household, classroom, school, and social or childcare contacts); $s_{j}$ was the relative susceptibility of individual$j$ (vs. children aged 0 years susceptibility); $r_{i}$ was the relative infectiousness of individual $i$ (vs. children aged 0 years infectiousness); $g_{i}$ and $g_{j}$ indicate the gender-related multiplier of individual $i$ and $j$, respectively; and $a_{i}$ was a multiplier of 1 if $i$ had asymptomatic disease and $w_{T}$ was the relative risk of transmission in the $T$ month, representing the seasonality of transmission.

**Intervention**

Current government or Centers for Disease Control and Prevention (CDC) recommended prevention and control measures are as follows: daily focus on improving hand hygiene of children followed by vaccination against HFMD as a preventive measure [39], children with HFMD should stay at home and away from school until them recover [40-42], classes were suspended for seven days if there are more than two classmates having an onset of HFMD in one classroom within one week [43], if a school or kindergarten has more than 10 cases, the school will be required to close for a period of 10 days [42]. Therefor, four single interventions, including three NPIs and vaccination, were considered in the model. Intervention measures are mainly defined by three parameters: whether to implement, application prerequisites, and (or) implementation intensity. They are as listed below:

Ⅰ: Isolation of symptomatic individuals. We assumed that sick (and infectious) individual will self-isolate or home quarantine when symptoms onset, meaning that exposure to sick individuals in the school context is likely to be truncated at an earlier time than would occur in the household.

Ⅱ: Class/Family quarantine [44, 45]. When there are more than two symptomatic individuals (application prerequisites) in the same family or class, the family or class will be isolated.

Ⅲ: Kindergarten closures. When there are more than 10 cases in the whole kindergarten within a week, or there are more than 2 cases in each of three classes (application prerequisites), the whole kindergarten will be suspended for 10 days.

Ⅳ: Vaccination. The vaccination scenario is simulated by setting two parameters, vaccine efficacy and vaccine coverage (implementation intensity). Vaccine efficacy indicates the percentage reduction in susceptibility of the vaccinated individual from the original base. This intervention was only available in the simulation of the EV-A71 strain.

**Search strategy and selection criteria**

A systematic review was undertaken following the PRISMA guidelines [46]. We searched for relevant publications pertaining to outbreaks of HFMD in a kindergarten-based or a school-based setting, in PubMed, Web of Science, Embase, China National Knowledge Infrastructure (CNKI), Sinomed, and WanFang Data, from Jan 1, 2010 to May 30, 2022. We applied the search string “(hand, foot and mouth*, OR HFMD, OR enterovirus, OR EV-A71, OR EV*, OR Coxsackievirus, OR CoxA*, OR CV*) AND (school OR kindergarten OR child care)”. For the literature on EV-A71, data from 2017 and later were removed to avoid the impact of vaccination on the results. No language or study design restriction was applied.

First, we screened titles and abstracts to identify potentially relevant articles. Second, the full-text review was applied to potentially relevant articles. We included publications with following conditions: (1) the outbreak occurred in a kindergarten, meeting the standard of cluster epidemic [47]; (2) specify the population structure (e.g., class and school sizes) of the kindergarten and the total number of students was more than 100; (3) there was a single index case; (4) incidence rate or number of cases were reported; (5) the serotype causing the outbreak can be identified and the implemented public health control measures were described. During the full-text review, the potential relevant cited references of the articles were also screened.

**Data extraction and model setup**

The collected literature was divided into three groups according to serotypes: EV-A71, CVA16 and other enterovirus (EVs). After classification, a random subset of 70% of literatures was used to create the training set for parameter estimation, and the remaining 30% of literatures were used as the test set for validation.

Data were extracted from included studies into a standard form (Table S8), including the following information: (1) class size, (2) the number of classes per grade, (3) the number of staffs, (4) month of outbreak occurrence, (5) duration of epidemic, (6) application prerequisites of school closure, (7) attack rates. And then we set up model of other fixed parameters and extract attack rates as the calibration targets. Seasonal relative transmission risks were adjusted for month of outbreak occurrence. Since the epidemic time reported in the literature was calculated from the onset of the first case, while the model was calculated from its exposure, and the outbreak was not considered to be over until there were no new cases for 10 consecutive days. In order to observe this complete process, the running time in the model is set to be 17 days longer than the epidemic time reported in the literature.

A total of 77 references were identified through systematically reviewing peer-review literatures. After literature screening, 23 records were included, including 12 articles related to EV-A71, 9 articles related to CVA16 and 2 articles related to other EVs (Fig. S2).

**Parameter estimation**

To address the challenge of directly sampling from complex posterior distributions, we employed the Sampling Importance Resampling algorithm (SIR) to estimate the baseline transmission rate ($\beta_{0}$). The algorithm approximates complex distributions by resampling from more easily sampled proposed distributions. The SIR algorithm involves three key steps. First, we assign to the baseline transmissibility a prior distribution U (0,0.1] (This range ensures that the single transmission probability is$\leq1$)，and uniformly sample from the prior distribution with an interval of 0.0001 to ensure broad exploration of parameter space. Each set of parameter simulations were run in duplicate 50 times. Second, we compare the difference between the simulated summary statistics (the mean attack rate of 50 simulations) and the observed one, using the relative Euclidean distance$d_{i}=\left| \frac{V_{i}-V_{iM}}{V_{iM}} \right|$, where $V_{i}$ and $V_{iM}$ denote the predicted and the observed attack rate of $i$th report, respectively. And then $d_{i}$ is converted to the importance sampling weight $L'\left( \theta_{i} \right)=exp\left( \frac{-1}{2}d_{i} \right)$ [48], where $\theta_{i}$ represents the input parameter set of $i$th report. Since each outbreak reported in the literature was independent with each other, the total sampling weight was the cumulative value of the sampling weight of a single outbreak $L=\prod_{i=1}^{n} L'\left( \theta_{i} \right)$. We select model simulations that satisfy $L$<*ϵ*, where *ϵ*, the particle acceptance rate, was chosen to pass 5% (50) of simulations into the accepted set. Third, resampling 300 times from the accepted set based on the sampling weights $L$, the resampling results were used as the posterior estimates, and the value with the largest sampling weight was used as the best estimates of the parameter. The best estimate and equal-tailed 95% Bayesian credible interval (BCI) of the posterior parameter sets are reported.

**Model validation**

The posterior estimation results are used as input parameters for the model, and we could obtain the corresponding predicted attack rate. To assess model performance, we compared whether the actual attack rate was within the 95% BCI of the predicted value. And we quantified predictive performance using the mean absolute prediction error (MAPE=$\frac{100\%}{n}\sum_{i=1}^{n} \frac{{|pred}_{i}-{obs}_{i}|}{{obs}_{i}}$) and correlation coefficient $r$between ${pred}_{i}$ and ${obs}_{i}$. The variables ${pred}_{i}$ and ${obs}_{i}$ are the $i$th predicted and observed value of a test set, respectively. MAPE quantifies the mean of the model's prediction error, with a lower MAPE indicating higher model accuracy; and *r* measures the linear relationship between actual and predicted attack rates. A correlation coefficient close to 1 indicates a strong positive correlation, suggesting that the predictions of the model are closely related to the trend of the actual data. If MAPE < 20% and *r* > 0.7, we regard the model fit as accurate, precise and suitable for simulations. Due to the limited literature pertaining to other EVs, we did not to perform model verification.

**Model calibration and model validation**

**Model parameters**

All model parameters are listed in Table S1.

**Seasonality of transmission**

HFMD in China is a recurring epidemic disease. Historical data show that the transmission rate of HFMD varies seasonally, with two peaks per year. The seasonal transmission was considered in our model, focusing on the relative risk of transmission $w_{T}$. We introduced trigonometric functions to describe periodicity and applied a quasi-Poisson regression to fit the incidence data for each month in China from 2010-2015 [49, 50]. To explore the qualitative characteristics of seasonally varying disease transmission and variable transmission rate, we estimated the relative transmission risk $w_{T}$ using the following equations:

$ln(N)$=$ln(P)+$ $b_{0}+b_{1} sin(\frac{}{6}T) +b_{2} cos(\frac{}{6}T) + b_{3} sin(\frac{}{3}T) + b_{4}cos\left( \frac{}{3}T \right)$(S1)

*λ*=${}_{0}w_{T}\propto ln(\frac{N}{P})$ (S2)

Where $N$ denotes the number of cases, $P$ is the number of exposed populations, and the expected monthly cases counts are obtained with formula (S1). *λ* represents seasonality of transmission, and ${}_{0}$ represents the average value for the year.

The results of quasi-Poisson regression are shown in Table S9, and the *R*^2^ of the model is 0.826, indicating a good fitting effect. We set the distribution of seasonal moderator of the model as a triangular distribution, in which the mode value was the predicted mean value, the minimum value was the lower limit of the 95% CI, and the maximum value was the upper limit of the 95% CI (Table S10). In each simulation, we randomly selected the distribution of the corresponding month.


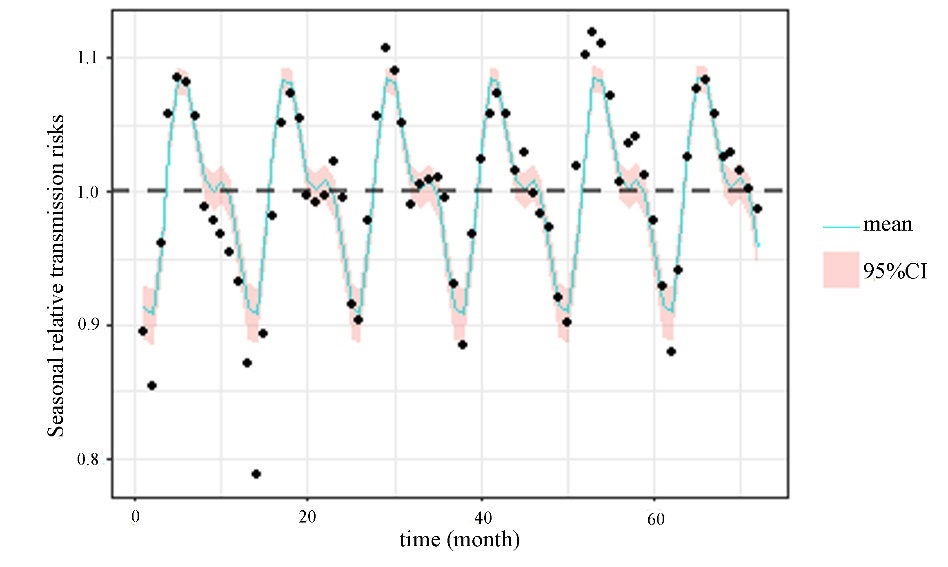


**Fig. S1. The fitting results of relative risk of transmission.**


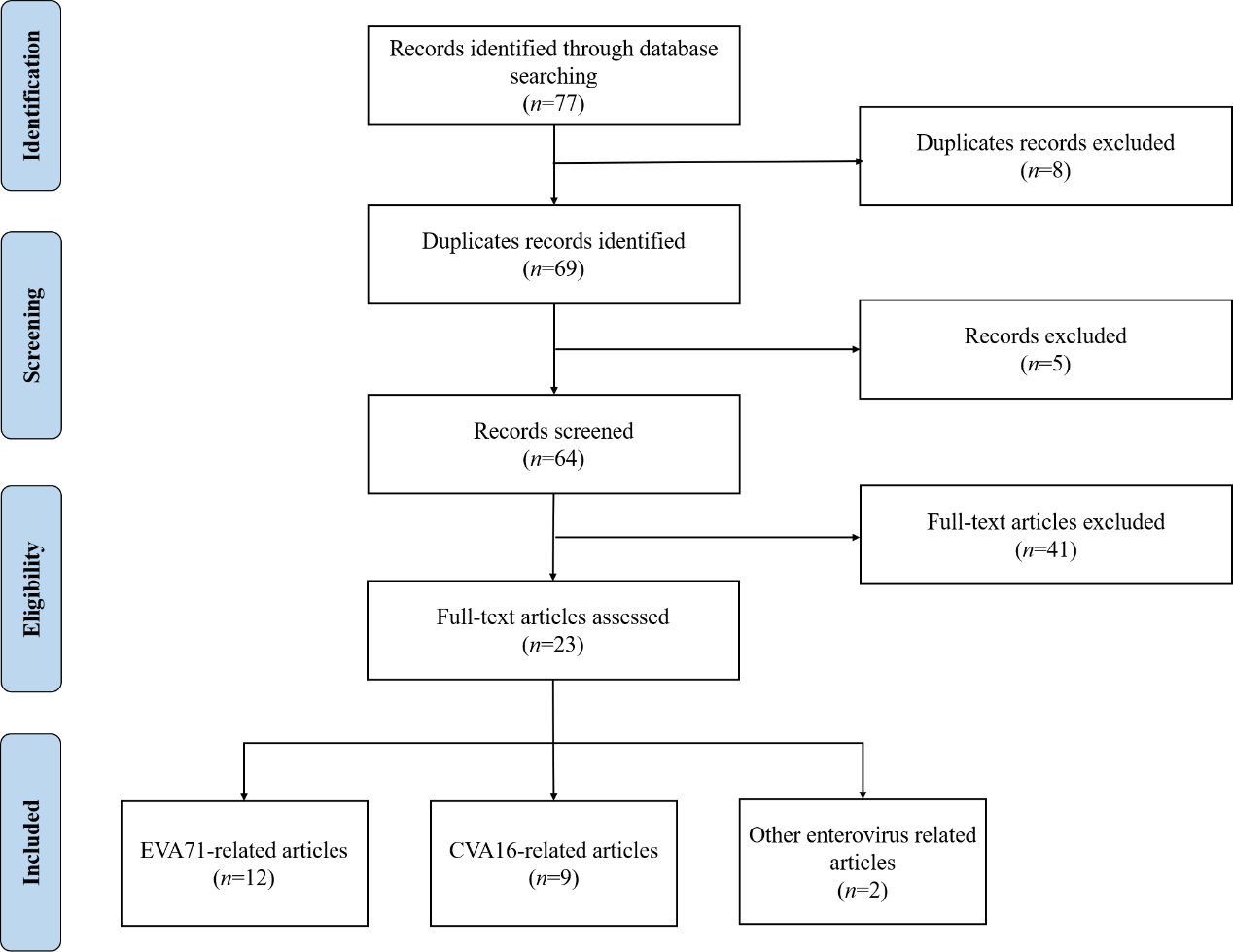


**Fig. S2. Data search and selection flow chart.**


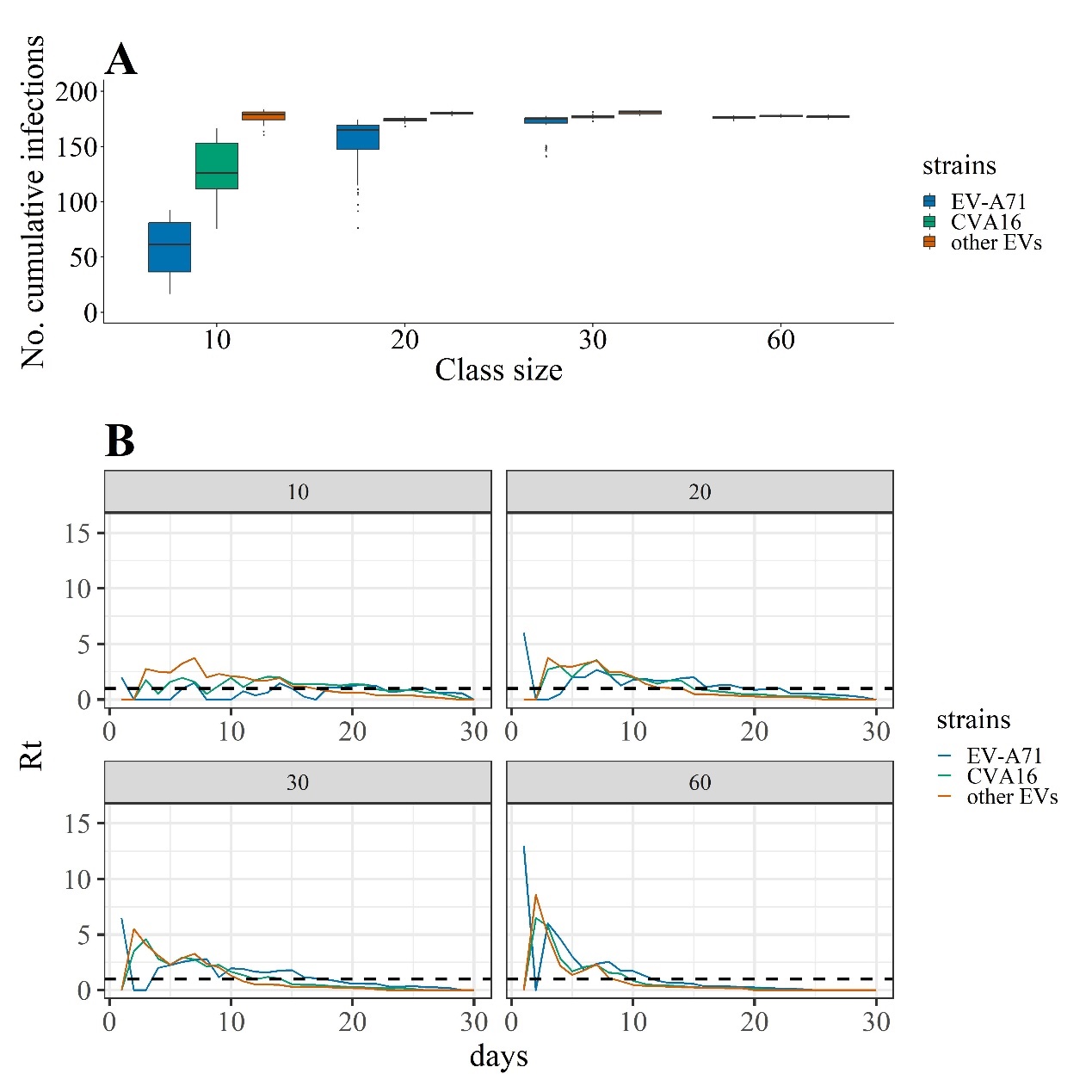


**Fig. S3. The transmission dynamics of hand, foot and mouth disease in small size kindergarten.** A showed the cumulative number of infections associated with EV-A71, CVA16 and other EVs. B showed the time series plot of the effective reproductions ($R_{t}$) per day with EV-A71, CVA16 and other EVs. Dashed line indicates $R_{t}$ =1.


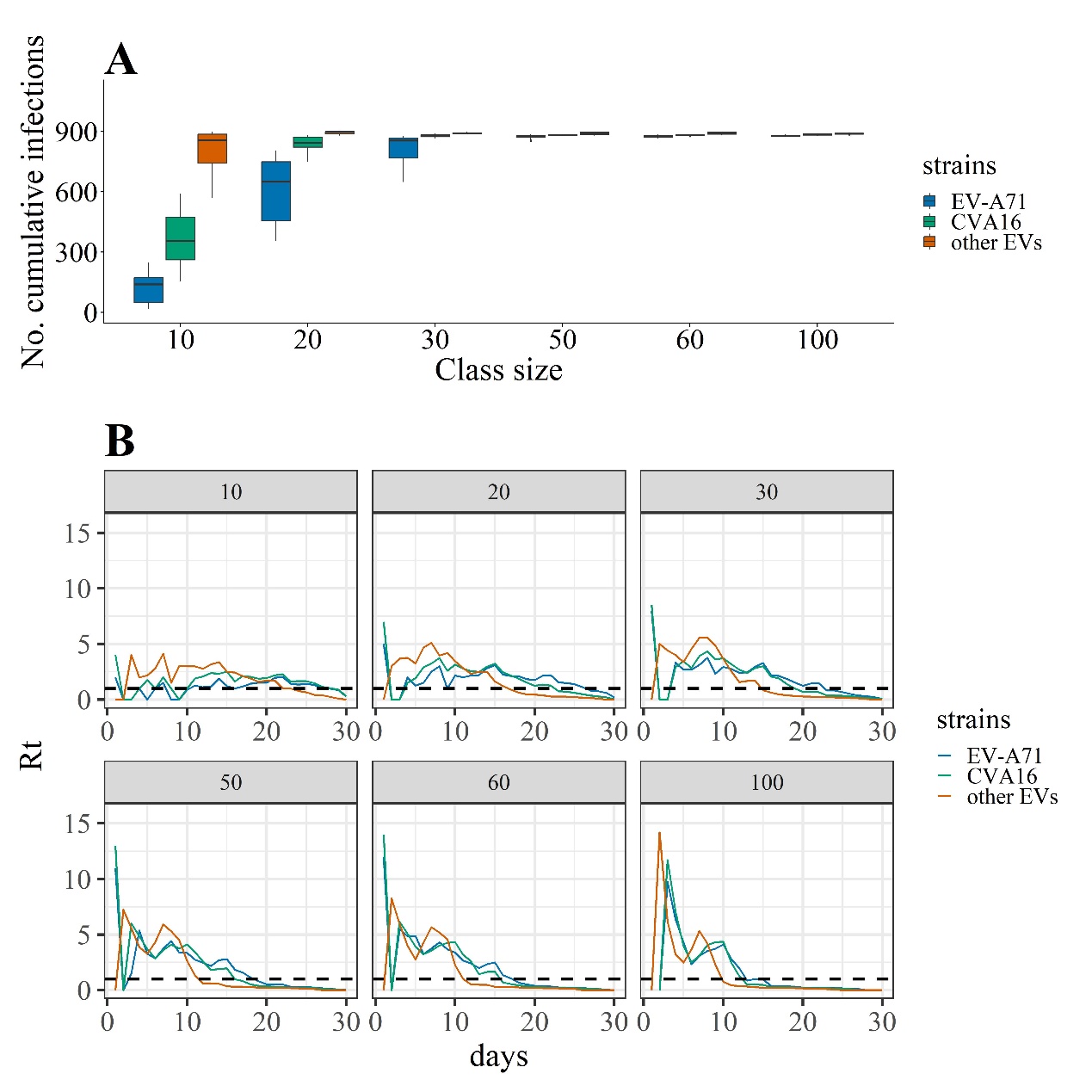


**Fig. S4. The transmission dynamics of hand, foot and mouth disease in large size kindergarten.** A showed the cumulative number of infections associated with EV-A71, CVA16 and other EVs. B showed the time series plot of the effective reproductions ($R_{t}$) per day with EV-A71, CVA16 and other EVs. Dashed line indicates $R_{t}$ =1.


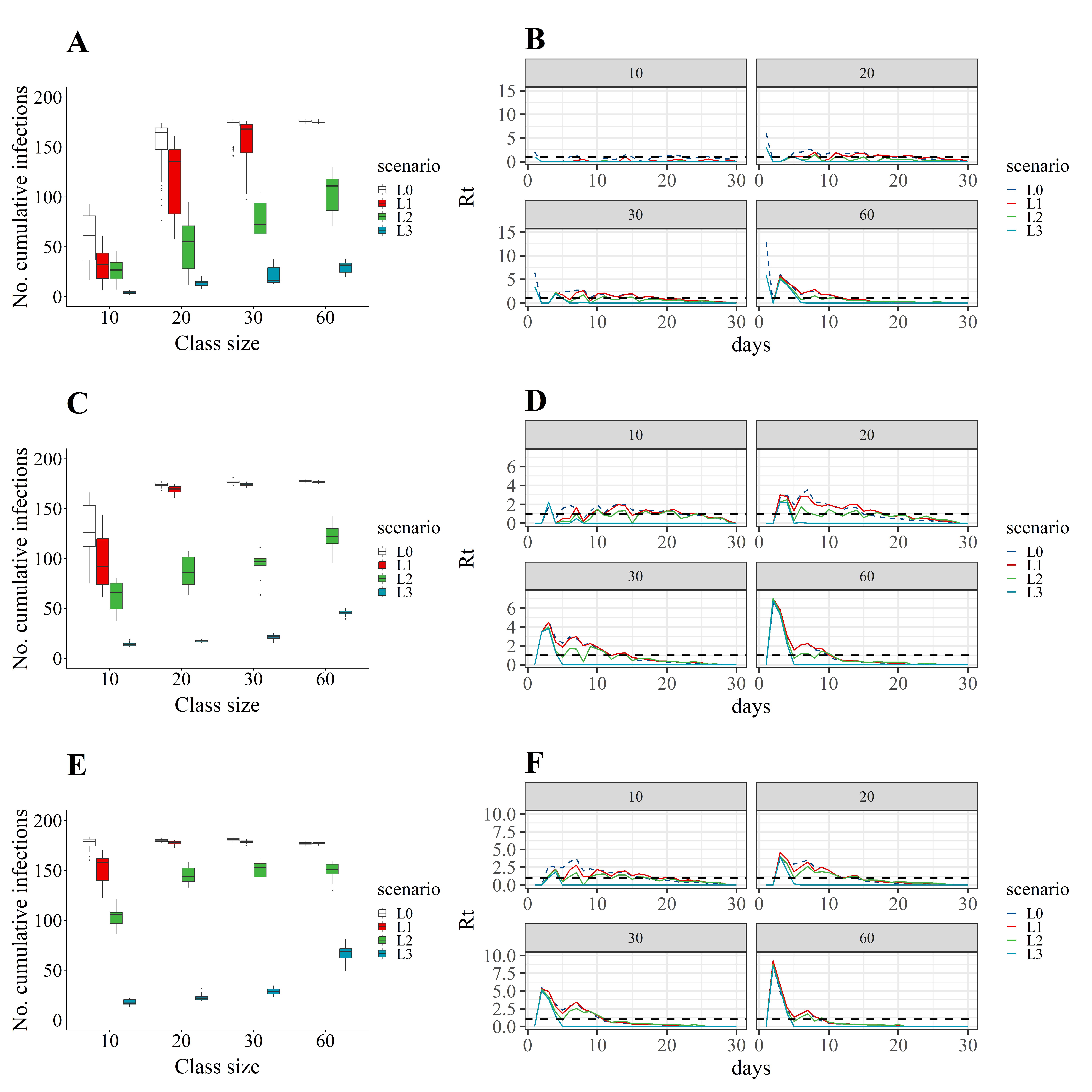


**Fig. S5. The transmission dynamics of hand, foot and mouth disease in small size kindergarten under three intervention strategies.** A, C, E showed the cumulative number of infections occurring at kindergarten, associated with EV-A71, CVA16 and other EVs, respectively. B, D and F showed the time series plot of the effective reproductions ($R_{t}$) per day with EV-A71, CVA16 and other EVs, respectively. Dashed line indicates $R_{t}$ =1.


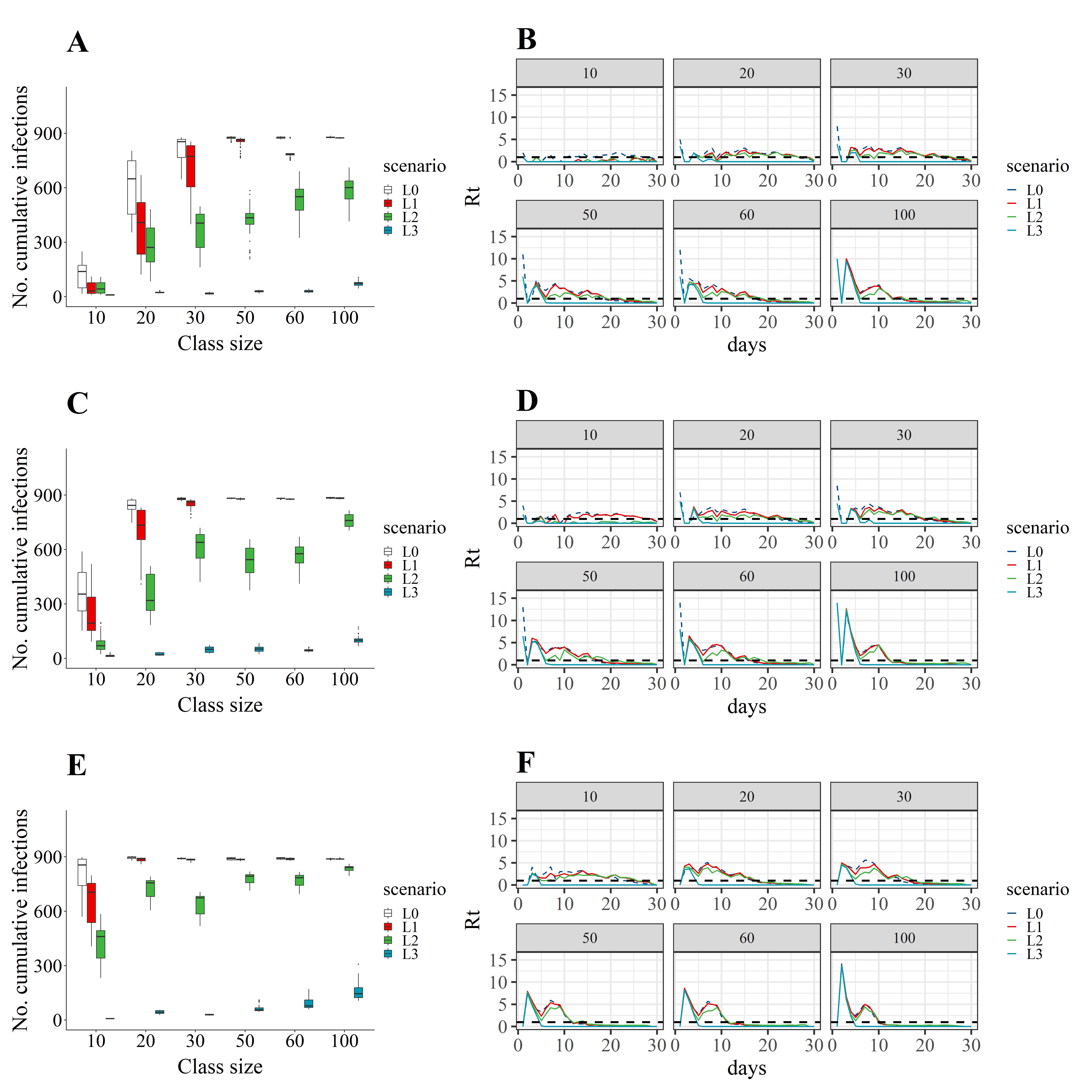


**Fig. S6. The transmission dynamics of hand, foot and mouth disease in large size kindergarten under three intervention strategies.** A, C, E showed the cumulative number of infections occurring at kindergarten, associated with EV-A71, CVA16 and other EVs, respectively. B, D and F showed the time series plot of the effective reproductions ($R_{t}$) per day with EV-A71, CVA16 and other EVs, respectively. Dashed line indicates $R_{t}$ =1.


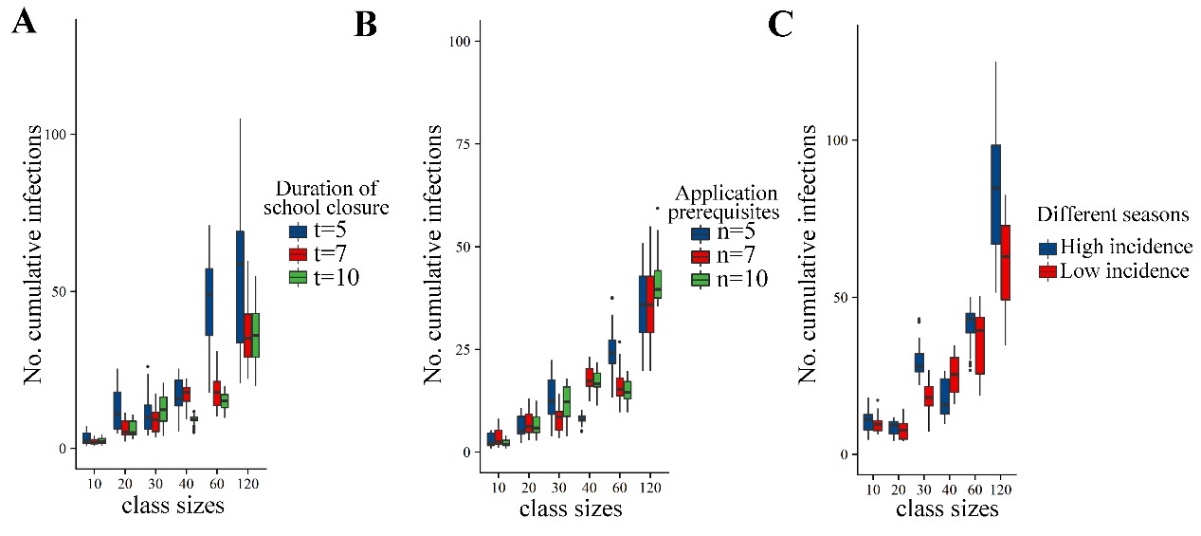


**Fig. S7. Cumulative number of infections under different parameter Settings.** (A)Different duration of school closure; (B) Different application prerequisites of school closure. Application prerequisites represent the number of symptomatic individuals in the kindergarten when the implementation of school closure; (C) Different seasons.

**Table S1. List of model parameters.**

|  | **Parameters** | **Description** | **Values** | **Sources** |
| --- | --- | --- | --- | --- |
| **Disease transmission** | $\beta_{0}$ | Basic transmission parameters | * | Parameter estimation. |
|  | $q_{class}$ | Transmission probability of classroom (vs. basic transmission parameters) | 1 |  |
|  | $q_{household}$ | Relative transmission probability of household contact (vs. classroom) | 1.7 | Published research [51]. |
|  | $q_{school}$ | Relative transmission probability of school contact (vs. classroom) | 0.25 | Published research [50, 52]. |
|  | $q_{social}$ | Relative transmission probability of social contact (vs. classroom) | 1 | Published research [53]. |
|  | $s_{j}$ | Age-specific susceptibility | 0 year: 1;  (0, 1] year: 1.96;  (1, 2] year: 3.14;  (2, 3] year: 4.14;  (3, 4] year: 4.14;  (4, 5] year: 4.39;  (5, 6] year: 4;  (6, 7] year: 3.12;  (7, 8] year: 2.22;  (8, 9] year: 1.95;  (9, 10] year: 1.96;  (10, 17] year: 1.2;  (17, +$\infty$) year: 0.5 | Published research [54]. |
|  | $r_{i}$ | Age-specific transmission | 1 | Published research [54]. |
|  | $g_{i}$ | Gender-related multiplier | Female:1; male:1.26 | Published research [55]. |
|  | $p_{aa}$ | Fraction of adults with asymptomatic disease | 0.99 | Published research [56]. |
|  | $p_{ac}$ | Fraction of child with asymptomatic disease | U [0.5-0.8] | Published research [57]. |
|  | $a_{i}$ | Multiplier on asymptomatic infection | 1 | Published research [58]. |
|  | $w_{T}$ | The relative risk of transmission in the $T$ month | Triangular distribution (Table S10) | Data Fitting. |
|  | $\tau_{es}$ | Time from exposure to onset of symptoms (incubation period) | $LogN (4.4,0.23)$ | Published research [59]. |
|  | $\tau_{is}$ | Time from the presence of infectiousness to the appearance of symptoms | $N(2,0.4)$ | Published research [60]. |
|  | $\tau_{inf}$ | The time when an individual has infectivity | $LogN (1.59,0.20)$ | Published research [61]. |
| **Intervention** | $\tau_{q}$ | Length of quarantine when someone is infectious | 10.00 | Published research [42]. |
|  | $p_{v}$ | Vaccine probabilities | 0.2,0.5,0.8 | Setting. |
|  | $p_{e}$ | Vaccine efficacy | 0.9 | Published research [62]. |

Parameter values are represented as probabilities or probability distributions.

*Indicated there are multiple values.

**Table S2. Results of parameter estimation**

| $\boldsymbol{\beta}_{\mathbf{0}}$ | **Prior distribution** | **Optimum estimate (95% BCI)** |
| --- | --- | --- |
| **EV-A71** | U(0, 0.1] | 0.0087 (0.0061, 0.0107) |
| **CVA16** | U(0, 0.1] | 0.0115 (0.0095, 0.0143) |
| **Other EVs** | U(0, 0.1] | 0.0190 (0.0146, 0.0219) |

Abbreviations: $\beta_{0}=$Basic transmission parameters. EV-A71 = enterovirus A71. CVA16 = coxsackievirus A16. Other EVs = other enterovirus.

**Table S3. Results of model validation.**

| **Serotypes** | **Actual value (%)** | **Predicted value (95% BCI) (%)** | **MAPE (%)** | $\boldsymbol{r}$ |
| --- | --- | --- | --- | --- |
| EV-A71 | 4.13 | 4.03 (2.46, 4.76) | 9.18 | 0.998 |
|  | 4.02 | 3.67 (2.16, 5.42) |  |  |
|  | 4.52 | 3.95 (2.67, 5.32) |  |  |
|  | 10.78 | 9.38 (5.36, 13.09) |  |  |
| CVA16 | 8.09 | 8.73 (6.87, 11.28) | 13.54 | 0.985 |
|  | 10.91 | 9.34 (6.63, 12.42) |  |  |
|  | 6.98 | 8.26 (6.81, 10.36) |  |  |

Abbreviations: EV-A71 = enterovirus A71. CVA16 = coxsackievirus A16.

**Table S4.** **Reduced cumulative infections by NPIs strategies in small kindergartens.**

|  |  | **EV-A71** |  | **CVA16** |  | **Other EVs** |
| --- | --- | --- | --- | --- | --- | --- |
| **Class sizes** | **Scenarios** | **Attributable percentage (%)** |  | **Attributable percentage (%)** |  | **Attributable percentage (%)** |
|  |  | **Median** (**95% BCI)** |  | **Median (95% BCI)** |  | **Median (95% BCI)** |
| **10** | **L0** | [Reference] |  | [Reference] |  | [Reference] |
|  | **L1** | 43.39 (22.80, 67.41) |  | 31.47 (13.50, 35.08) |  | 14.32 (12.03, 21.15) |
|  | **L2** | 54.20 (35.25, 71.01) |  | 64.73 (42.19, 59.93) |  | 41.30 (40.12, 44.68) |
|  | **L3** | 91.52(82.53, 94.10) |  | 91.63 (82.56, 91.93) |  | 92.18 (90.33, 92.50) |
| **20** | **L0** | [Reference] |  | [Reference] |  | [Reference] |
|  | **L1** | 22.16 (8.92, 49.60) |  | 8.28 (-0.47, 7.10) ^a^ |  | 1.61 (0.54, 2.82) |
|  | **L2** | 57.31 (46.91, 85.04) |  | 53.50 (39.07, 64.18) |  | 19.16 (17.18, 23.76) |
|  | **L3** | 92.45 (89.66, 94.40) |  | 95.79 (90.91, 92.63) |  | 90.46 (89.82, 91.00) |
| **30** | **L0** | [Reference] |  | [Reference] |  | [Reference] |
|  | **L1** | 5.13 (-0.04, 24.77) ^a^ |  | 3.84 (-0.99, 3.98) ^a^ |  | 0.46 (-0.18, 1.71) |
|  | **L2** | 57.31 (41.61, 68.54) |  | 57.89 (36.50, 63.72) |  | 16.39 (12.67, 20.90) |
|  | **L3** | 91.88 (83.70, 93.43) |  | 90.63 (89.49, 92.81) |  | 88.24 (87.49, 89.11) |
| **60** | **L0** | [Reference] |  | [Reference] |  | [Reference] |
|  | **L1** | -0.37 (-2.77, 2.60) ^a^ |  | 0.53 (-2.43, 3.54) ^a^ |  | -0.57 (-1.62, 0.65) |
|  | **L2** | 37.40 (24.86, 57.45) |  | 41.09 (21.09, 44.89) |  | 16.55 (14.52, 18.19) |
|  | **L3** | 85.93 (84.32, 90.64) |  | 90.52 (79.44, 83.60) |  | 73.41 (72.75, 75.08) |

Abbreviations: EV-A71 = enterovirus A71. CVA16 = coxsackievirus A16. Other EVs = other enterovirus. L0 = baseline with no intervention; L1 = only isolation of symptomatic individuals; L2 = combination of isolation of symptomatic individuals and class and family quarantine; L3 = combination of three NPIs, including isolation of symptomatic individuals, class and family quarantine and kindergarten closure (governmental recommended NPIs strategies in child care setting).

^a^Indicates the difference was not statistically significant.

**Table S5. Reduced cumulative infections by NPIs strategies in large kindergartens.**

| **Class sizes** | **Scenarios** | **EV-A71** |  | **CVA16** |  | **Other EVs** |
| --- | --- | --- | --- | --- | --- | --- |
|  |  | **Attributable percentages (%)** |  | **Attributable percentages (%)** |  | **Attributable percentages (%)** |
|  |  | **Median (95%BCI)** |  | **Median 95%BCI** |  | **Median 95%BCI** |
| **10** | **L0** | [Reference] |  | [Reference] |  | [Reference] |
|  | **L1** | 60.47 (28.97, 80.10) |  | 37.27 (6.70, 53.11) |  | 22.52 (17.87, 27.17) |
|  | **L2** | 59.56 (26.12, 81.85) |  | 76.53 (63.98, 91.42) |  | 49.12 (47.29, 52.09) |
|  | **L3** | 91.12 (57.24, 95.02) |  | 97.20 (89.19, 97.93) |  | 99.08 (99.05, 99.11) |
| **20** | **L0** | [Reference] |  | [Reference] |  | [Reference] |
|  | **L1** | 36.25 (19.08, 63.61) |  | 16.35 (7.70, 45.76) |  | 1.68 (0.96, 2.06) |
|  | **L2** | 56.66 (44.73, 71.01) |  | 61.77 (43.80, 75.25) |  | 17.36 (14.38, 24.90) |
|  | **L3** | 96.47 (92.45, 97.41) |  | 97.93 (96.73, 98.31) |  | 96.23 (95.57, 96.74) |
| **30** | **L0** | [Reference] |  | [Reference] |  | [Reference] |
|  | **L1** | 12.53 (3.58, 41.31) |  | 3.29 (1.70, 14.30) |  | 1.82 (1.41, 2.41) |
|  | **L2** | 53.52 (45.17, 73.86) |  | 28.17 (19.41, 50.32) |  | 25.36 (24.28, 34.13) |
|  | **L3** | 98.24 (97.70, 98.47) |  | 95.28 (93.36, 97.36) |  | 97.43 (97.30, 97.57) |
| **50** | **L0** | [Reference] |  | [Reference] |  | [Reference] |
|  | **L1** | 2.76 (1.24, 13.52) |  | 0.45 (-0.85, 1.82) ^a^ |  | 0.82 (0.53, 1.55) |
|  | **L2** | 51.04 (36.47, 74.71) |  | 39.23 (27.61, 57.17) |  | 12.18 (11.35, 15.18) |
|  | **L3** | 97.22 (96.68, 98.10) |  | 95.46 (92.79, 97.82) |  | 95.33 (94.56, 95.75) |
| **60** | **L0** | [Reference] |  | [Reference] |  | [Reference] |
|  | **L1** | 10.82 (-0.33, 15.85) ^a^ |  | 1.0148 (-0.07, 2.65) ^a^ |  | 0.26 (-0.35, 0.72) ^a^ |
|  | **L2** | 36.27 (20.73, 60.11) |  | 35.55 (24.77, 51.72) |  | 12.96 (11.06, 16.26) |
|  | **L3** | 97.41 (95.88, 97.89) |  | 96.28 (94.62, 97.06) |  | 93.55 (90.64, 94.20) |
| **100** | **L0** | [Reference] |  | [Reference] |  | [Reference] |
|  | **L1** | 0.17 (-1.08, 1.32) ^a^ |  | 0.18 (-1.11, 1.85) ^a^ |  | -0.49 (-0.94, -0.08) |
|  | **L2** | 32.42 (20.32, 46.62) |  | 13.89 (7.70, 19.81) |  | 6.60 (5.97, 7.81) |
|  | **L3** | 93.92 (91.39, 95.37) |  | 91.73 (86.73, 94.21) |  | 88.09 (84.92, 89.94) |

Abbreviations: EV-A71 = enterovirus A71. CVA16 = coxsackievirus A16. Other EVs = other enterovirus. L0 = baseline with no intervention; L1 = only isolation of symptomatic individuals; L2 = combination of isolation of symptomatic individuals and class and family quarantine; L3 = combination of three NPIs, including isolation of symptomatic individuals, class and family quarantine and kindergarten closure (governmental recommended NPIs strategies in child care setting).

^a^Indicates the difference was not statistically significant.

**Table S6. Attributes of agents.**

| **Type** | **ID** | **HH ID** | **HH type** | **Age** | **Sex** | **CN** | **Grade** |
| --- | --- | --- | --- | --- | --- | --- | --- |
| **student** | √ | √ | √ | √ | √ | √ | √ |
| **siblings** | √ | √ | √ | √ | √ | × | × |
| **parents** | √ | √ | × | × | × | × | × |
| **teacher** | √ | × | × | × | × | √ | × |
| **staff** | √ | × | × | × | × | × | × |

Abbreviations: ID = identifier. HH ID = household id. HH type = household type. CN = class number. √Indicates that the presence of the attribute for the corresponding agent type. ×Indicates that the absence of the corresponding attribute.

**Table S7. Information about the three contact layers.**

| **Contact layers** | | **Contacts** | **Selection method** | **Time of contact** |
| --- | --- | --- | --- | --- |
| **Kindergarten** | Within the classroom | Classmates, teachers | Everyone in the classroom | Monday - Friday |
|  | Inter the classroom | Classmates, teachers, staff | Randomly select 20 people in the school | Monday - Friday |
| **Household** | Inside the household | Parents, siblings | All family members | Monday - Sunday |
| **Place for public activities** | Outside the household | Other families | Randomly match 2 families | Monday - Sunday |

**Table S8. Summary of EV-A71-related and CVA16-related survey data of HFMD outbreaks.**

|  | **Group^#^** | **Month** | **Class sizes** | **No. classes** | **No. children** | **No. teachers** | **No.other staff** | **Total number of people** | **Application prerequisites (days)** | **Duration (days)** | **Attack rate (%)** |
| --- | --- | --- | --- | --- | --- | --- | --- | --- | --- | --- | --- |
| **EV-A71** | 1 | 4 | 30 | 9 | 270 | 18 | 11 | 299 | 10 | 8 | 3.70 |
|  | 1 | 5 | 30 | 12 | 360 | 24 | 33 | 417 | 12 | 11 | 4.47 |
|  | 1 | 9 | 28 | 6 | 168 | 12 | 6 | 198 | 8 | 14 | 4.48 |
|  | 1 | 4 | 44 | 3 | 132 | 6 | 5 | 143 | 9 | 8 | 8.51 |
|  | 1 | 11 | 35 | 9 | 315 | 18 | 11 | 344 | 18 | 18 | 7.94 |
|  | 1 | 6 | 31 | 6 | 186 | 12 | 4 | 196 | 10 | 5 | 3.47 |
|  | 1 | 6 | 28 | 24 | 672 | 48 | 45 | 765 | 6 | 6 | 1.67 |
|  | 1 | 5 | 27 | 6 | 162 | 12 | 6 | 180 | 11 | 9 | 7.30 |
|  | 2 | 5 | 30 | 12 | 360 | 24 | 14 | 398 | 10 | 11 | 4.13 |
|  | 2 | 9 | 28 | 9 | 252 | 18 | 25 | 295 | 12 | 9 | 4.02 |
|  | 2 | 3 | 40 | 3 | 120 | 6 | 3 | 129 | 15 | 13 | 10.78 |
|  | 2 | 5 | 30 | 12 | 369 | 24 | 27 | 411 | 13 | 13 | 4.52 |
| **CVA16** | 1 | 5 | 23 | 9 | 207 | 18 | 9 | 234 | 35 | 21 | 15.09 |
|  | 1 | 10 | 55 | 3 | 165 | 6 | 0^#^ | 171 | 5 | 6 | 9.76 |
|  | 1 | 6 | 29 | 6 | 174 | 12 | 11 | 197 | 6 | 9 | 8.97 |
|  | 1 | 4 | 32 | 18 | 576 | 36 | 32 | 644 | 12 | 8 | 2.14 |
|  | 1 | 10 | 38 | 6 | 228 | 12 | 9 | 249 | 39 | 32 | 15.22 |
|  | 1 | 11 | 25 | 3 | 75 | 6 | 0 | 81 | 10 | 14 | 12.04 |
|  | 2 | 6 | 34 | 9 | 306 | 18 | 17 | 341 | 22 | 13 | 8.09 |
|  | 2 | 11 | 33 | 6 | 198 | 12 | 7 | 217 | 18 | 15 | 10.91 |
|  | 2 | 3 | 25 | 6 | 150 | 12 | 9 | 171 | 12 | 8 | 6.98 |
| **Other EVs** | 1 | 5 | 34 | 6 | 204 | 12 | 16 | 232 | 9 | 11 | 4.15 |
|  | 1 | 12 | 27 | 9 | 243 | 18 | 22 | 283 | 12 | 11 | 4.29 |

Abbreviations: EV-A71 = enterovirus A71. CVA16 = coxsackievirus A16. Other EVs = other enterovirus.

**Table S9. The result of quasi-Poisson regression.**

|  | **Estimate (95% CI)** | **Std.Error** | ***t*** | ***P* value** | ***R^2^*** |
| --- | --- | --- | --- | --- | --- |
| $\boldsymbol{b}_{\boldsymbol{0}}$ | -9.192 (-9.276, -9.110) | 0.042 | -217.641 | < 0.001* | 0.826 |
| $\boldsymbol{b}_{\boldsymbol{1}}$ | -0.253 (-0.371, -0.136) | 0.060 | -4.217 | < 0.001* |  |
| $\boldsymbol{b}_{\boldsymbol{2}}$ | -0.743 (-0.856, -0.635) | 0.056 | -13.166 | < 0.001* |  |
| $\boldsymbol{b}_{\boldsymbol{3}}$ | -0.426 (-0.535, -0.319) | 0.055 | -7.751 | < 0.001* |  |
| $\boldsymbol{b}_{\boldsymbol{4}}$ | 0.234 (0.129,0.338) | 0.053 | 4.371 | < .001* |  |

*Indicates *P*< 0.05.

**Table S10.** **The triangular distribution parameter of relative risk of transmission.**

| Month | 1 | 2 | 3 | 4 | 5 | 6 | 7 | 8 | 9 | 10 | 11 | 12 |
| --- | --- | --- | --- | --- | --- | --- | --- | --- | --- | --- | --- | --- |
| Mode | 0.91 | 0.9 | 0.95 | 1.03 | 1.09 | 1.09 | 1.05 | 1.01 | 1 | 1.01 | 1.01 | 0.96 |
| Min | 0.89 | 0.88 | 0.93 | 1.02 | 1.08 | 1.08 | 1.03 | 0.99 | 0.99 | 1 | 0.99 | 0.94 |
| Max | 0.92 | 0.91 | 0.96 | 1.05 | 1.11 | 1.11 | 1.06 | 1.02 | 1.02 | 1.03 | 1.02 | 0.97 |
